# DGR-MAPPO: Enhancing Multi-Agent Cooperative Exploration in Unknown Environments with Distance-Aware Communication and Global Average Pooling

**DOI:** 10.3390/s26154882

**Published:** 2026-08-03

**Authors:** Chufang Wang, Xiai Chen, Aoqi Shen, Jiongkun Yang

**Affiliations:** School of Mechanical and Electrical Engineering, China Jiliang University, No. 258 Xueyuan Street, Hangzhou 310018, China; p24010854107@cjlu.edu.cn (C.W.); shenaoqiyue@163.com (A.S.); s23010811031@cjlu.edu.cn (J.Y.)

**Keywords:** deep reinforcement learning, multi-agent, collaborative exploration, unknown environment, MAPPO

## Abstract

To address the challenges of local observation limitations, difficulties in global collaboration, and low exploration efficiency encountered in multi-agent collaborative exploration of unknown environments, this paper introduces a multi-agent proximal policy optimization (MAPPO) algorithm that incorporates distance awareness and gated recurrent unit optimization. The study constructs a composite observation space that integrates global maps with local perceptions. The stability of feature extraction is improved through the introduction of a global average pooling layer, while a distance-based dynamic communication mechanism is developed to facilitate map information sharing among neighboring agents. Experimental results demonstrate that this approach significantly reduces the path overlap rate in untrained complex maps, markedly enhances exploration coverage and training convergence speed, and confirms its effectiveness in improving the collaborative efficiency of multi-agents and their adaptability to varying environments.

## 1. Introduction

With the rapid advances in artificial intelligence and robotics, the perception and decision-making capabilities of autonomous agents in complex environments have emerged as a key research frontier. In particular, exploration tasks in unknown or hazardous areas-such as planetary surface surveying [1], post-disaster search and rescue, industrial facility inspection, and large-scale warehouse logistics management-pose an urgent demand for efficient and robust autonomous exploration systems. Although traditional single-agent systems perform well in structured environments, they exhibit inherent limitations in terms of sensing coverage, decision-making capacity, and task completion efficiency when confronted with large-scale, dynamic, and highly uncertain settings. To address these challenges, multi-agent systems (MAS) [2] have been introduced; through cooperation and division of labor among multiple agents, MAS can substantially improve execution parallelism, spatial coverage, and overall system robustness, demonstrating significant application potential.

However, achieving efficient multi-agent collaborative exploration faces a series of core challenges. The primary issue lies in how to generate strategies for each agent in a high-dimensional, partially observable dynamic environment that are both conducive to individual decision-making and promote global collaboration [3]. Traditional centralized planning methods often rely on precise environmental models and global communication, making it difficult to adapt to the actual situation of real-time changes and limited communication [4]. While a fully decentralized approach can enhance the flexibility and scalability of the system, it is prone to behavioral conflicts among agents, overlapping exploration areas, and resource waste. Therefore, how to strike a balance between distributed decision-making and global collaboration is a key scientific issue in the field of multi-agent exploration.

In recent years, the advent of Deep Reinforcement Learning (DRL) [5,6] has introduced a novel approach for addressing complex collaborative problems. This methodology allows agents to derive optimization strategies directly from high-dimensional perceptual data through trial-and-error interactions with their environment, independent of precise modeling. Extending this framework to the multi-agent context, referred to as multi-agent Reinforcement Learning (MARL) [7], has paved the way for enhanced coordination among multiple agents and the optimization of long-term cumulative returns [8]. Notably, algorithms based on the Actor-Critic architecture and the Centralized Training with Decentralized Execution (CTDE) paradigm have garnered significant attention. These algorithms effectively leverage global information for strategy optimization during the training phase while relying solely on local observations to make independent decisions during execution. This dual capability strikes a favorable balance between the demands of collaboration and the feasibility of deployment.Although existing studies have confirmed the effectiveness of MARL in collaborative tasks, several critical issues remain that must be addressed urgently for its application in collaborative exploration of unknown environments [9,10]. These issues include: designing efficient observation representations that allow agents to accurately infer the global environmental structure and the state of collaborative objects from local perceptions; establishing effective coordination among agents through limited communication in the absence of a central controller to minimize redundant exploration; and enhancing the adaptability, training stability, and generalization capabilities of the algorithm in dynamic and complex environments.

In response to these challenges, this paper investigates a multi-agent collaborative exploration method grounded in deep reinforcement learning. The objective of this study is to design and validate a comprehensive solution. The primary contributions are as follows:

1. A DGR-MAPPO (Distance-aware GAP Recurrent MAPPO) algorithm that integrates local observation with a dynamic communication mechanism is proposed. This approach introduces a distance-based information-sharing strategy, enabling agents within the communication range to exchange information and incrementally update their respective maps. Consequently, this method effectively mitigates observation limitations, significantly enhances exploration coverage, and reduces path overlap.
2. A joint representation method was developed that integrates local positional features with global state information. A corresponding feature extraction network was designed, employing global average pooling to effectively capture the global semantic information from high-dimensional features. This approach reduces model complexity while preserving the ability to express features. Additionally, a GRU network was incorporated to effectively capture the dynamic evolution of the agent’s state during collaborative exploration, thereby enhancing training stability and increasing the average reward.
3. A grid simulation environment designed for multi-agent collaborative exploration was developed for the MAPPO algorithm framework. This environment enables agents to perceive both global and local contexts through dual-channel image fusion observations. Additionally, it facilitates the simultaneous perception of an agent’s overall exploration state and local environmental details, which include radar data, obstacle distribution, and multi-agent position coding. Consequently, this setup enhances the collaborative exploration capabilities of the multi-agent system.

The rest of the paper is organized as follows: Section 2 provides an overview of the multi-agent cooperative exploration problem and related literature. Section 3 introduces DGR-MAPPO, the method proposed in this paper for efficient cooperative exploration of robots. Section 4 reports the experimental results, comparing DGR-MAPPO with frontier-based exploration methods and discussing the results and their broader implications. Finally, Section 5 concludes the paper, highlighting key contributions and outlining directions for future research.

## 2. Background

### 2.1. Problem Definition

**Figure 1:**
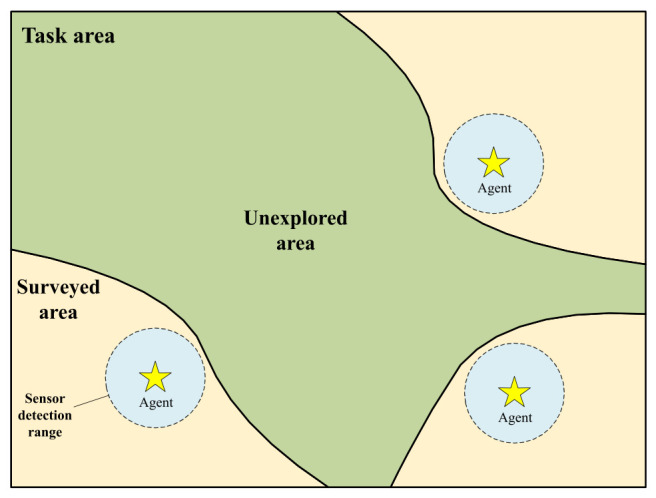
Multi-agent collaborative exploration of the scene.

The schematic diagram of multi-agent collaborative exploration is shown in Figure 1. The multi-agent cooperative exploration problem can be formulated as a dynamic graph coverage problem. Let G=(V,E) be an undirected graph, where *V* represents the set of vertices (corresponding to discretized grid cells) and *E* represents the set of edges (representing the effective movement paths between cells). Consider a set $A=\{a_{1},a_{2},\ldots ,a_{N}\}$ of *N* isomorphic mobile agents. Each agent $a_{i}$ traverses the entire graph *G* while avoiding collisions. Let FOV (field of view) be the local sensing range of agent $a_{i}$, where $r_{\mathrm{obs}}$ is the observation radius measured in grid cells. Agents exchange map data with other agents within their communication radius $r_{\mathrm{comm}}$.

Each vertex v∈V in the graph has three states: unexplored, explored (idle), or obstacle. When formulating the multi-agent exploration problem as a graph optimization problem, the goal is to find a set of collaborative strategies $\Pi =\{\pi_{1},\pi_{2},\ldots ,\pi_{N}\}$ that optimize a selected objective function, such as minimizing the total number of time steps *T* required to cover the entire graph or minimizing the total distance traveled by all agents, while ensuring that the following constraints are met:

1. Full coverage constraint: All reachable vertices must eventually be visited and marked as explored by at least one agent.
2. Obstacle and collision avoidance constraints: Agents must not enter obstacle vertices, and at any time *t*, no more than one agent may occupy the same vertex (i.e., vertex conflicts are prohibited). Additionally, edge conflicts (e.g., two agents swapping positions along the same edge simultaneously) should be avoided if required by the model.
3. Local information constraint: Each agent’s decision-making is based solely on its local field of view (within observation radius $r_{\mathrm{obs}}$) and information received from other agents within its communication radius $r_{\mathrm{comm}}$.

### 2.2. Related Work

#### 2.2.1. Non-Learning-Based Approaches

Exploration strategies that utilize frontier and information approaches are essential for addressing exploration tasks in unknown environments. In early traditional single-agent autonomous exploration schemes, the focus was typically on boundary points or regions characterized by higher uncertainties within a grid graph, facilitating the gradual development of a comprehensive understanding of the environment. Once the frontier points are identified, a graph traversal algorithm will be employed. Breadth-First Search (BFS) [11] and Depth-First Search (DFS) [12] are two fundamental algorithms utilized in graph traversal. BFS explores graphs through hierarchical expansion, ensuring the identification of the shortest path in unweighted graphs. In contrast, DFS delves as deeply as possible along a single path within the graph. In boundary-based exploration methods, Yamauchi introduced a pioneering boundary-based automatic exploration algorithm, which has significantly influenced the field of autonomous exploration [13]. This algorithm identifies the boundary between Free and Unknown areas, designating it as the target point for subsequent exploration efforts. Building on this foundational work, a series of automatic exploration methods have emerged. In 2006, Freda and Oriolo proposed a boundary-based probabilistic exploration strategy [14], which introduced a probabilistic model to more accurately capture environmental uncertainties, moving beyond a simplistic grid occupancy model. This advancement enables agents to more effectively discern the boundaries between known and unknown regions. In 2010, Wettach and Berns introduced the strategy of dynamic boundary exploration [15]. This approach employs a dynamic frontier point update mechanism, allowing agents to continuously adapt to changes in their environment, which renders it suitable for complex and variable indoor settings. In 2011, Mobarhani et al. developed a boundary exploration method utilizing histographs [16]. This technique enhances information representation by constructing an occupation histogram of the environment. The histogram captures the occupation status of various spatial regions, including known, unknown, and obstacle areas, thereby facilitating efficient analysis and selection of frontier regions. Recently, numerous methods have focused on accelerating boundary detection speed. Keida and Kaminla proposed the Fast Boundary Detection algorithms, namely the Wavefront Frontier Detector (WFD) and the Fast Frontier Detector (FFD) [17]. Additionally, Umari introduced the Rapidly-Exploration Random Tree method [18] for edge detection.

In the study of multi-agent exploration, Yamauchi introduced a boundary-based exploration method within multi-agent systems. This approach enables each agent to maintain a local map during exploration and independently select boundary points as targets for investigation [19]. The primary goal of this method is to gather more information about unknown areas while maximizing environmental coverage. Although agents can share a global map to comprehend explored regions, conflicts or congestion may arise due to their independent actions in the absence of a coordination mechanism. This lack of coordination can negatively impact overall exploration efficiency and resource utilization. Bautin et al. proposed an enhanced exploration method in which each agent assesses its relative ranking based on the traveling distance to various boundary points [20]. Based on this ranking, each agent is assigned to a specific boundary point for visitation and exploration.

Traditional algorithms demonstrate strong performance in structured and static environments; however, their limitations are evident. These algorithms depend significantly on precise modeling of the environment. When faced with dynamic obstacles, partial observability, or inaccuracies in maps due to sensor noise, their performance declines sharply. Additionally, in large-scale or high-dimensional environments, the number of frontier points can increase exponentially, resulting in excessive computational overhead. More critically, these methods do not possess the capacity to learn from experience and cannot adaptively modify their exploration strategies based on task characteristics, rendering them inflexible in complex and ever-evolving real-world scenarios.

#### 2.2.2. Learning-Based Approaches

MARL represents a significant frontier in artificial intelligence, focusing on the core issue of multiple autonomous agents learning collaborative or competitive strategies through interactions within a shared environment. The theoretical foundation of MARL extends the single-agent Markov Decision Process (MDP) to Markov games in multi-agent contexts [21]. However, this extension introduces several fundamental challenges, rendering MARL research considerably more complex than that of single-agent reinforcement learning.

The primary challenges encountered in MARL include non-stationarity, credit assignment, scalability, and partial observability [22]. The issue of non-stationarity arises because the learning processes of individual agents dynamically alter the environment experienced by other agents, thereby violating the fundamental assumptions necessary for the convergence of single-agent reinforcement learning algorithms. The credit assignment problem involves the equitable distribution of global rewards achieved by the team among the contributions of each individual agent in cooperative settings, which is essential for effective learning. The scalability challenge pertains to the increase in computational and communication overheads associated with algorithms as the number of agents expands, raising concerns about potential declines in performance. Partial observability indicates that, in real-world scenarios, agents typically access only local information about the environment rather than the complete global state, further complicating the learning process [23].

To tackle these challenges, researchers have devised a range of mainstream methodologies. Notably, “Centralized Training with Decentralized Execution” (CTDE) has emerged as the predominant paradigm in cooperative multi-agent reinforcement learning (MARL) [24,25]. This approach effectively distinguishes between the training and execution phases: during training, a centralized critic, equipped with global information, delivers stable learning signals and mitigates the problem of credit allocation. In contrast, during execution, each agent independently makes decisions based solely on its local observations, thereby preserving the system’s distributed nature and facilitating practical deployment. The CTDE framework has given rise to a variety of representative algorithms. For instance, MADDPG (Multi-Agent Deep Deterministic Policy Gradient) effectively addresses the non-stationarity issue by employing a centralized commentator for each agent, which has access to the states and actions of all agents [26]. QMIX utilizes the concept of value function factorization, merging individual Q values into global Q values via a mixed network while imposing monotonicity constraints to maintain strategy consistency. Consequently, it has demonstrated remarkable performance in benchmark evaluations, including the StarCraft Multi-Agent Challenge (SMAC) [27].

However, some current CTDE methods encounter challenges in multi-agent collaborative exploration tasks. MADDPG, a derivative of the DDPG framework, operates as an off-policy deterministic policy gradient method. The Critic and Actor networks of MADDPG are susceptible to training instability issues in sparse reward settings, exhibit sensitivity to hyperparameters, and demonstrate subpar convergence [28]. Additionally, MADDPG generates deterministic strategies, which initially lack adequate exploration capabilities during training, often causing all agents to adopt similar behaviors. Consequently, this leads to extensive path overlap and diminished exploration efficiency. Value decomposition approaches like QMIX face constraints related to monotonicity and struggle to represent intricate collaboration strategies.

Multi-Agent Proximal Policy Optimization (MAPPO) is a prominent algorithm grounded in policy gradient methods within the Centralized Training with Decentralized Execution (CTDE) framework, demonstrating considerable advantages. MAPPO incorporates the trust region optimization concept from single-agent Proximal Policy Optimization (PPO) [29] and employs an approximate objective function with clipping to regulate policy updates. This approach enhances the robustness and stability of the training process [30], making it particularly well-suited for exploration tasks in environments characterized by sparse rewards [31,32].

In recent years, deep reinforcement learning has rapidly advanced for multi-agent exploration in unknown environments. As reviewed by Nguyen et al. [33], multi-agent deep reinforcement learning faces persistent challenges in non-stationarity, partial observability, scalability, and coordination, motivating task-specific designs. MARVEL [34] incorporates visibility constraints into its policy for large-scale, limited-field-of-view multi-robot exploration. D-MARL [35] integrates dynamic communication with an augmented action space to improve coverage; however, its learned differentiable message generation incurs substantial overhead and does not enforce a hard communication-range constraint. Learning-based communication schemes such as communication-aware graph neural networks [36] and graph-modeling-based communication learning [37] enable differentiable message routing but typically assume a fully connected graph, limiting their applicability to range-constrained deployments [38]. These methods either presume global connectivity, rely on computationally intensive learned message passing, or are task-specific, leaving on-demand map sharing and redundant-path avoidance in unknown environments insufficiently addressed. To bridge this gap, this work introduces a lightweight distance-triggered communication mechanism that exchanges local map information only when agents are within a predefined range, preserving information-sharing benefits while bounding overhead for realistic deployment.

The application of MAPPO has significantly influenced several key domains. In autonomous driving, Zhao et al. introduced the AM-MAPPO algorithm, which utilizes an action masking mechanism to address the safety coordination challenges faced by unmanned vehicles merging onto highway ramps [39]. Leandro Parada et al. developed an avoidance and coordination framework based on MAPPO for high-priority traffic scenarios involving the passage of emergency vehicles [40]. Regarding collaborative tasks among unmanned aerial vehicles (UAVs), Zhou et al. proposed the BCTD-MAPPO algorithm in 2025, which integrates behavioral cloning and temporal difference learning for collaborative target tracking in dense obstacle environments [41]. This approach effectively mitigates the risk of training converging to local optima in high-dimensional observation spaces by employing an expert trajectory initialization strategy and refining the strategy through MAPPO, thereby enhancing the continuous tracking capability for highly maneuverable evading targets. Furthermore, Pan et al. introduced MS-MAPPO in 2023, specifically tailored for multi-agent search and rescue operations in entirely unknown environments [42]. In the context of adversarial unmanned systems, Pu et al. proposed the Velocity Domain MAPPO (VD-MAPPO) in 2024, designed for unmanned surface vehicle (USV) formations engaged in perimeter defense adversarial tasks [43].

The MAPPO algorithm has undergone systematic verification across various complex and collaborative domains, showcasing its stability and scalability. This evidence underscores MAPPO’s reliability as a universal framework for multi-agent collaboration. Our approach builds upon the MAPPO algorithm and incorporates three significant enhancements for multi-agent collaborative exploration tasks. First, we have developed a dual-channel image fusion observation space, which improves the agents’ joint perception of both the global context and the local environment, while mitigating the computational burden associated with high-dimensional observations. Second, we implemented a lightweight transformation of the policy network architecture by substituting the traditional fully connected layer with a global average pooling layer. This modification substantially decreases the number of model parameters while effectively preserving spatial feature information, thereby improving the training stability and generalization capability of the agents. Finally, we introduced a distance-triggered dynamic communication mechanism, integrating it with the GRU memory module to facilitate the exchange of critical state information only when necessary. This enhancement effectively reduces the path conflict rate and improves regional coverage efficiency.

## 3. Method

In this work, we address the multi-agent cooperative exploration problem by developing a method that integrates deep reinforcement learning based on MAPPO with an explicit communication mechanism. The proposed approach is specifically designed to coordinate multiple agents operating under limited communication ranges and partial observability. Our approach relies on decentralized control, and robots make decisions based on their local observations.

This paper presents a comprehensive overview of the MAPPO method designed for collaborative exploration, addressing six fundamental aspects: (i) the MAPPO model framework; (ii) the reward structure; (iii) the observation space; (iv) the communication mechanism; (v) the gated recurrent unit; and (vi) the algorithm framework and flowchart. We elaborate on the design of a local observation space derived from the agent’s surrounding environment and clarify how information exchanged with nearby agents through the communication mechanism is utilized.

### 3.1. MAPPO

MAPPO is a multi-agent near-end policy optimization deep reinforcement learning algorithm that builds upon the PPO framework to tackle collaborative tasks involving multiple agents. As a prominent algorithm within the centralized training with decentralized execution (CTDE) framework, MAPPO employs a centralized Critic network alongside a decentralized Actor network, thereby improving the efficiency of collaboration and the stability of learning in multi-agent systems.

Prior to the introduction of MAPPO, IPPO was commonly employed in multi-agent reinforcement learning tasks. IPPO serves as a direct extension of single-agent PPO, treating each agent as an independent learner and applying PPO individually to update each agent’s policy. This method does not necessitate centralized training or global information, which enhances its simplicity and scalability. Nonetheless, its limitations are apparent. Since each agent is trained independently, IPPO fails to fully leverage global state information or the actions of other agents, and it does not effectively tackle the credit assignment problem in multi-agent environments.

MAPPO enhanced the IPPO framework to address its shortcomings. Within the Actor network, the original policy optimization method remains intact. During the training phase, a centralized Critic network is incorporated, which utilizes global information for value assessment. This Critic network estimates the global value function by considering the global state and the actions of all agents, typically employing the Temporal Difference (TD) method for estimation. The TD algorithm integrates dynamic programming and Monte Carlo approaches, updating the value function estimation based on the immediate rewards from the current state and the subsequent state. At each step, it relies solely on the current estimated value, eliminating the need to wait for the entire sequence to conclude before performing cumulative return calculations. The complete training procedure of MAPPO, including advantage estimation and the updates of the centralized Critic and decentralized Actor networks, is summarized in Algorithm 1.
**Algorithm 1:** MAPPO (Multi-Agent Proximal Policy Optimization)
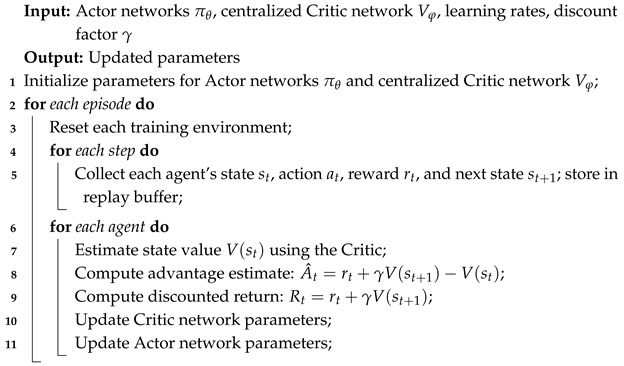


### 3.2. Reward Function

The design of the agents’ reward function is a critical component of multi-agent reinforcement learning, as it directly affects learning performance and the resulting behavioral patterns. In this study, the agents’ reward function r(t) is computed as follows:

(1) $$r\left(t\right)=r_{e}\left(t\right)+r_{c}\left(t\right)+r_{s}\left(t\right)$$

Among these, $r_{e}\left(t\right)$ represents the exploration reward, which incentivizes the agent to discover previously unexplored areas. Each time the agent successfully explores a new grid cell, it receives a reward of 1. $r_{c}\left(t\right)$ signifies the collision penalty, which aims to mitigate conflicts between agents and obstacles. Each time an agent collides, it receives a penalty of −10. The term $r_{s}\left(t\right)$ denotes the step penalty, which serves to limit the agent’s action efficiency and prevent unnecessary step consumption during the exploration process. The agent incurs a penalty of −0.2 for each step taken.

(2) $$r_{e}\left(t\right)=\left\{\begin{array}{cc} 1 & \mathrm{if}\;\mathrm{exploring}\;\mathrm{the}\;\mathrm{unknown}\;\mathrm{grid}\\ 0 & \mathrm{else} \end{array}\right.$$

(3) $$r_{c}\left(t\right)=\left\{\begin{array}{cc} -10 & \mathrm{if}\;\mathrm{agent}\;\mathrm{collision}\\ 0 & \mathrm{else} \end{array}\right.$$

(4) $$r_{s}\left(t\right)=-0.2$$

With this reward design, the exploration reward, step penalty, and collision penalty complement each other, enabling agents to acquire the following behavioral patterns during training: (i) active exploration: agents prioritize previously uncovered regions to maximize overall exploration efficiency; (ii) efficient behavior: the step penalty discourages unnecessary movements, thereby improving learning efficiency; and (iii) coordination and conflict avoidance: the collision penalty encourages agents to coordinate with other agents in complex environments, avoid collisions, and maintain safe operation.

### 3.3. Observation Space

**Figure 2:**
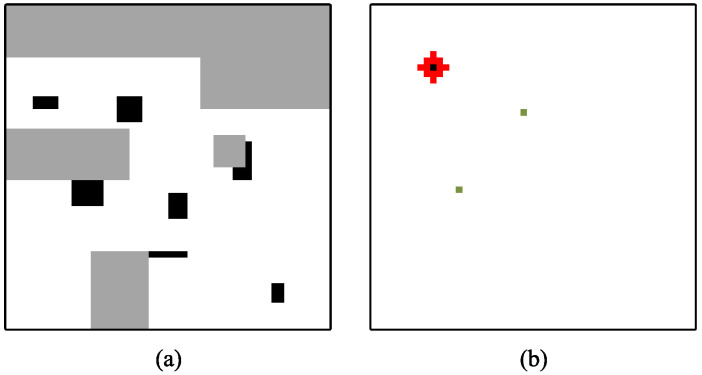
Two different feature maps of different channels. (**a**) The first feature map (black: obstacles, gray: unexplored areas); (**b**) the second feature map (green dot: agent’s current position, red area: perception range).

The design of the agents’ observation space is fundamental to enabling effective environmental perception and interaction, and it is also a key factor influencing algorithmic performance. In this study, the observation space is characterized as a two-dimensional feature map measuring 50×50×2, where ‘2’ denotes the number of input feature channels. This design integrates both global and local information and allows feature extraction via convolutional neural network (CNN), thereby enhancing the representational capacity of the model. The detailed design is as follows:

The first feature map represents the local observation of each agent, i.e., a partial view of the environment perceived within each agent’s individual observation range under the MAPPO framework. Consistent with the CTDE (Centralized Training with Decentralized Execution) paradigm, each agent operates solely on its own local observation during both training and execution, without directly accessing any global map. This local observation map encodes three types of information: explored areas, unexplored areas, and the locations of static obstacles within the agent’s current field of view. As illustrated in Figure 2a, white regions denote explored areas, gray regions indicate unexplored areas, and black regions correspond to static obstacles. During training and execution, these three states are encoded as numerical values 0, −1, and 1, respectively, to facilitate network input processing and to distinguish environmental states. This encoding is both intuitive and efficient, providing a clear informational basis for agents’ decision-making.

The second feature map encodes the agent’s own observations as well as the positions of other agents. As shown in Figure 2b, black indicates the current agent’s position, green denotes the positions of the other agents, and the red region represents the agent’s sensing range, which is intended to emulate the observation footprint of a radar sensor in real-world scenarios. To differentiate among region types, a value-shifting scheme is applied to the observable area during training and execution. Specifically, within the sensor range, the obstacle cells are encoded as 1.5 and free cells as 0.5, while the positions of other agents are encoded as −0.5. This encoding enables the agent to distinguish different environmental elements and to make more accurate decisions.

### 3.4. Communication Mechanism

In single-agent reinforcement learning, agents typically have access to a comprehensive view of the entire environment, enabling them to optimize strategies and decision-making effectively. However, multi-agent reinforcement learning introduces additional complexity as agents must account for the states of other agents and their environmental effects. Consequently, the observation space expands significantly, posing challenges in acquiring and processing global observation data directly.

Therefore, to improve the efficacy of local observations, agents can exchange critical information, including explored areas and obstacle locations, via communication mechanisms. This exchange mitigates the issue of limited information resulting from local observations. Such information sharing can occur through explicit communication methods, such as message-passing networks, or through implicit information fusion techniques, such as attention mechanisms, thereby facilitating more effective collaboration.

**Figure 3:**
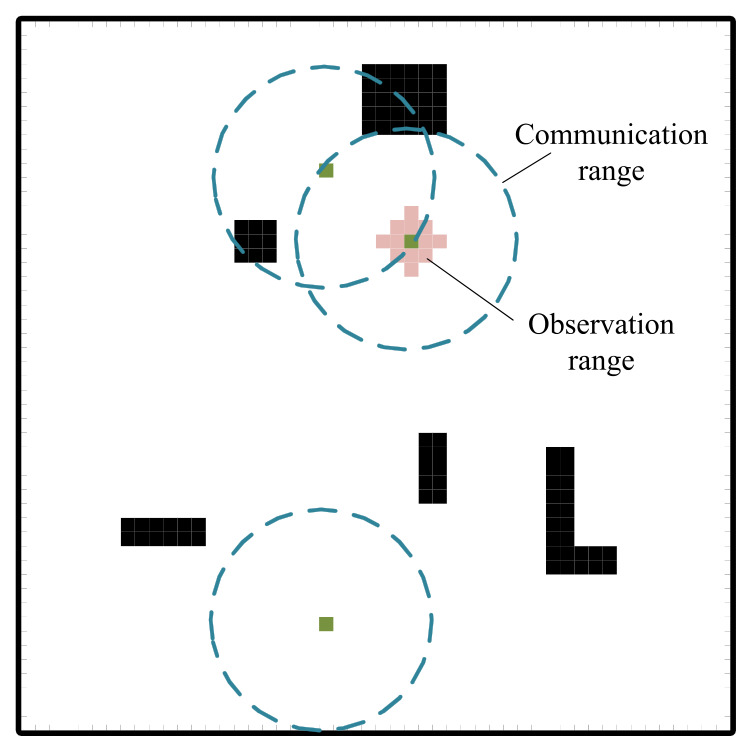
Communication mechanism diagram.

The agent’s access is limited to the local map information it generates during execution, preventing it from observing the states of other agents. To address this information isolation, the experiment incorporates a communication mechanism among agents. When the distance between two or more agents falls within the predefined communication range, they automatically share their respective local map data. This process enables the agent to acquire relevant information regarding the exploration areas of other agents in real time, thereby enhancing its environmental awareness. As illustrated in Figure 3, the area delineated by the blue dotted line signifies the communication range of the agents. Within this range, agents are able to exchange information and progressively update their maps. Conversely, the area represented by the pink region denotes the observation range, within which agents can explore the map.

### 3.5. Recurrent Neural Network

**Figure 4:**
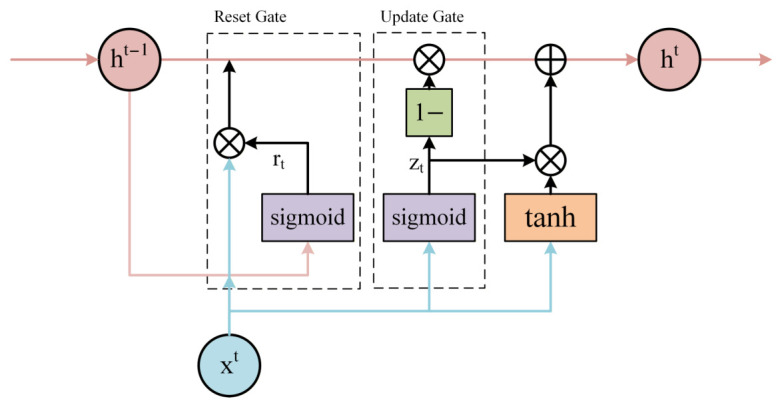
Architectural Diagram of Gated Recurrent Unit.

The Gated Recurrent Unit (GRU) [44] has been optimized for the problems of complex computation and large number of parameters in LSTM networks, which can greatly improve the training efficiency. Compared with LSTM, GRU simplifies the gating mechanism by merging the original forgetting gate, input gate and output gate into two gating units: reset gate and update gate, and removes the long-term memory chain. Figure 4 below is a schematic diagram of the gated circulation unit.

The calculation formula for the reset door $r_{t}$ as follows:

(5) $$r_{t}=sigmoid\left(h_{t-1}\cdot W_{r}+x_{t}\cdot U_{r}+b_{r}\right)$$

The calculation formula for the update gate $z_{t}$ as follows:

(6) $$z_{t}=sigmoid\left(h_{t-1}\cdot W_{z}+x_{t}\cdot U_{z}+b_{z}\right)$$

This method incorporates a gated recurrent unit (GRU), which allows the agent to more effectively leverage historical information, optimize decision-making processes, and improve exploration performance. The GRU addresses the challenges of long-term dependency in recurrent neural networks (RNN) and mitigates the complexities associated with training long short-term memory networks (LSTM) that have numerous parameters. Consequently, this approach facilitates more efficient modeling of time series information across various tasks.

### 3.6. Algorithm Framework and Flowchart

**Figure 5:**
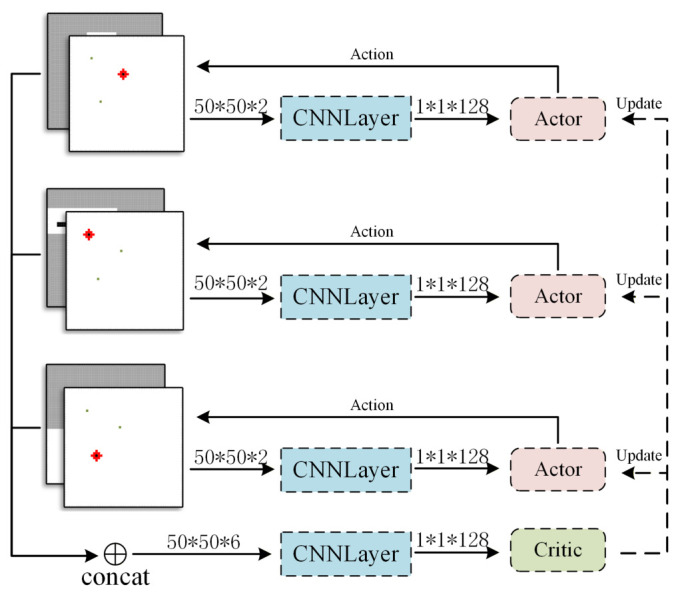
Algorithm framework. Red area: perception range.

Figure 5 illustrates the training and decision-making process of the agent within the MAPPO framework, utilizing local observations. This framework restricts each agent to access only local environmental information, represented by 50×50×2 feature maps. These two-channel feature maps encompass local map data and the agent’s positional information. The local map data includes details on three states: explored areas, unexplored areas, and obstacle locations. Given the limited observational range of each agent, they are unable to directly access the global state of the entire environment. Consequently, they must depend on policy learning to facilitate reasonable decision-making despite the presence of incomplete information.

**Figure 6:**
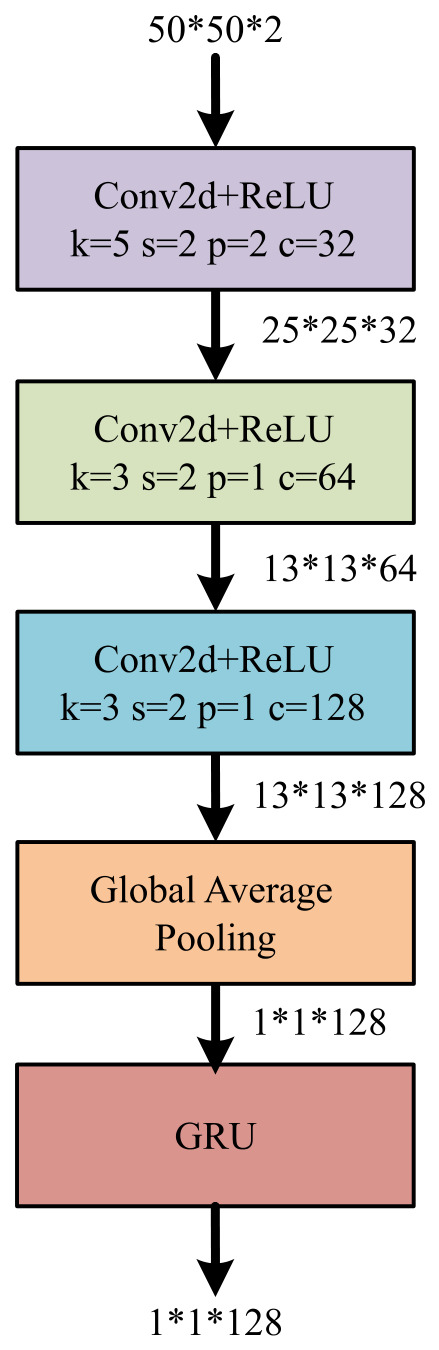
CNN architecture incorporating global average pooling.

The network structure of the CNN Layer is shown in the following Figure 6.

During the model training phase, each agent first inputs the perceived local environmental information into the multi-scale visual feature encoding module. This module features a multi-level convolutional structure and comprises three feature extraction units, each configured with heterogeneous convolutional kernels (5×5, 3×3, 3×3). Each unit integrates two-dimensional convolutional operators with ReLU activation functions. Following multi-scale spatial feature fusion, global average pooling is employed to compress the high-dimensional features, resulting in a 128-dimensional observation feature code. This encoded vector is subsequently input into the Policy generation network (Actor), where a deep neural network calculates the optimal behavioral policy based on the current environmental state. The output of the policy network corresponds to a set of five actions, which include four planar movement directions (up, down, left, and right) and a stationary state. This design ensures that the agent can adapt flexibly to environmental changes, facilitating efficient autonomous exploration and decision-making.

To establish a global environmental assessment mechanism, the system employs multi-dimensional channel fusion technology, which integrates the perception data from each agent along the channels to create a composite observation tensor of dimensions 50×50×6. The construction of this tensor relies on the local observation information from three agents, each with a feature map size of 50×50×2. By superimposing these observations in the channel dimension, a unified global representation is formed. This tensor serves as the input for the central evaluation network, where it undergoes feature extraction via a convolutional neural network akin to the policy network. Ultimately, this process yields a scalar evaluation value that represents the global environmental value. This evaluation parameter not only indicates the quality of the current overall system status but also provides a foundation for gradient updates during the strategy optimization process.

## 4. Experimental Results and Performance Analysis

### 4.1. Introduction to the Simulation Environment

**Figure 7:**
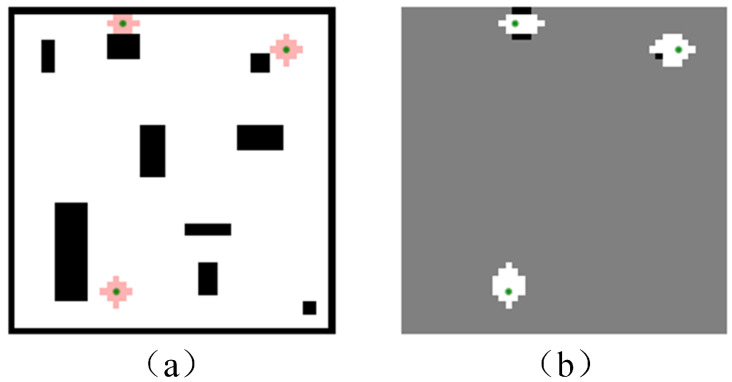
(**a**) Grid simulation environment (black: obstacles, gray: unexplored areas, green dot: agent’s current position, red area: perception range); (**b**) multi-agent exploration schematic diagram. Black: obstacles, white: explored areas, gray: unexplored areas, green dot: agent’s current position.

The simulation environment utilized in this experiment is structured as a grid, designed to replicate dynamic interactions within a multi-agent collaborative exploration task. This grid comprises a total of 50×50 cells, with each cell representing a spatial position that reflects various environmental states. Figure 7a illustrates the layout of the grid environment, where white areas denote explored regions, black areas indicate obstacles, the green area marks the agent’s current position, and the red area signifies the agent’s perception range. Each agent’s objective is to explore as many unexplored regions as possible while avoiding collisions with obstacles and conflicts with other agents. Figure 7b depicts the exploration of the task area, with gray regions representing areas yet to be explored. The agent incrementally expands the explored area through movement and observation, while also receiving feedback from the environment. This design seeks to evaluate the effectiveness of multi-agent collaboration and decision-making strategies in complex settings. Agents must not only devise efficient action paths but also consider coordination and cooperation with other agents to maximize exploration efficiency and minimize collisions.

### 4.2. Experiment Settings

This experiment is developed based on the deep learning framework PyTorch (version 2.7.0) and adopts the MAPPO algorithm combined with the CTDE architecture to simulate and simulate the task of multi-agent collaborative exploration in a grid environment. Through the powerful neural network construction and training functions of PyTorch, the efficient development of the feature extraction, action decision-making and value assessment modules of the agent has been achieved. The Actor and Critic optimizer algorithms adopt the commonly used Adam algorithm. Table 1 below is the set of parameters for my experiment.

**Figure 8:**
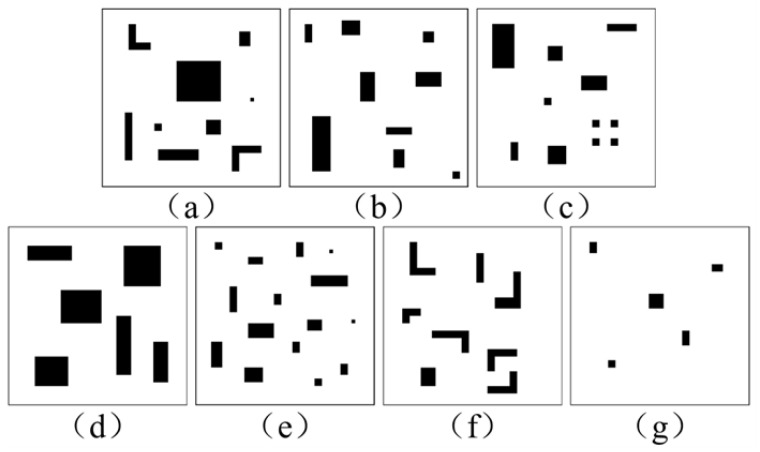
Seven types of training environments. (**a**–**g**) represent seven types of training environments, encompassing a range of configurations from dense to sparse obstacles, with the primary shapes being rectangular and L-shaped.

In the experiment, three agents were deployed to simultaneously explore a grid environment, thereby simulating a multi-agent collaboration scenario. To expedite the training process and enhance sampling efficiency, the experiment utilized a strategy of running three environments in parallel. The agents concurrently sampled data across these multiple environments for training purposes. This parallel design significantly enhances sample collection efficiency and mitigates the risk of model overfitting associated with a single environment, thereby improving the agents’ generalization capabilities. To ensure the comparability of the experiments, all three utilized the same network architecture, which featured CNN-based feature extraction and employed global average pooling to reduce computational complexity and enhance generalization. This architecture minimizes the number of parameters while preserving spatial information, thereby improving training stability and accelerating convergence speed. To simulate the various types and sizes of obstacles that may be present in a factory environment, a diverse grid diagram setting was incorporated during the training process. Seven distinct grid diagrams with varying layouts were utilized, as illustrated in Figure 8 below. These grid maps encompass a range of configurations, from dense to sparse obstacles, with the primary shapes being rectangular and L-shaped. This design ensures that the agent can develop robust exploration strategies in complex and variable scenarios, while also mitigating the overfitting problem associated with exploration limited to a single map. Additionally, the agent’s position is randomly initialized each time the environment is reset. This approach aims to enhance data diversity, yielding a broader range of training data from different initial conditions, thereby improving the model’s generalization ability and adaptability.

**Figure 9:**
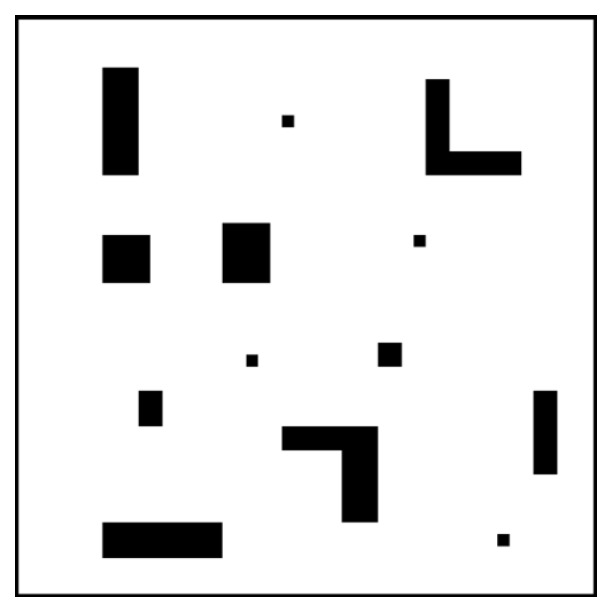
Untrained baseline environment.

To further quantify the adaptability and generalization ability of each method, Figure 8d,e, and the untrained Figure 9 were selected as the basis for comparative analysis: Figure 8d,e are mainly used to evaluate the adaptability of the agent when facing different sizes of obstacles, where (d) represents the large obstacle environment (denoted as Map I), and (e) represents the complex small obstacle environment (denoted as Map II). Figure 9 serves as the untrained benchmark environment (denoted as Map III), and is used to test the generalization ability of each model in a new environment.

### 4.3. Comparative Experiment

This paper presents a comparison between the proposed DGR-MAPPO algorithm and a multi-agent collaborative exploration method, referred to as the BFS method. The communication range of DGR-MAPPO is 50. The BFS method integrates Breadth-First Search with a nearest frontier-first strategy. Initially, the system employs a greedy strategy based on the Manhattan distance, sequentially allocating frontier points to the nearest idle agent. Concurrently, the BFS algorithm is utilized to determine the shortest contactless path from each agent to its designated frontier point. Subsequently, the agent proceeds along the planned path without updating the leading points. Upon reaching its target frontier point, the system re-scans the current map to refresh the frontier point set, assigns a new target frontier point, and plans a new path for the agent. This iterative process continues until the map exploration is complete. This approach effectively combines optimal path planning via BFS with task allocation driven by frontier points, enabling collaborative coverage exploration by multiple agents while maintaining collision-free paths. The complete exploration procedure is summarized in Algorithm 2.
**Algorithm 2:** BFS-Based Multi-Agent Collaborative Exploration
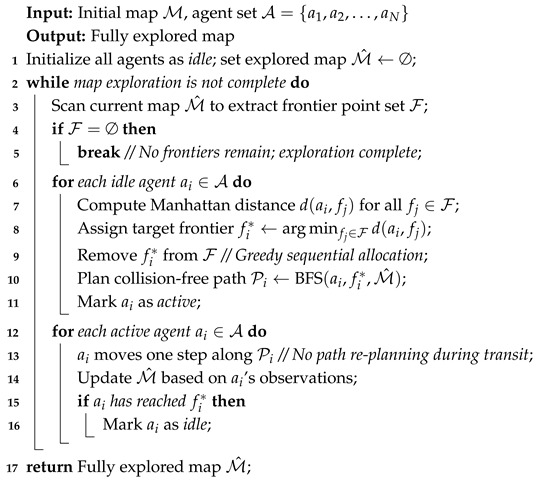


Table 2, Table 3 and Table 4 provide detailed comparisons of the exploration performance of two different algorithm schemes (DGR-MAPPO, BFS) in various map environments, covering key indicators such as the lowest exploration rate, the highest exploration rate, the average exploration rate, the number of convergence steps, and the exploration variance.

**Figure 10:**
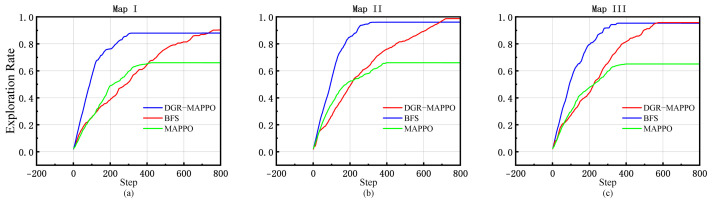
Comparison chart of exploration rates between MAPPO, DGR-MAPPO and BFS in different maps. (**a**) Map I; (**b**) Map II; (**c**) Map III.

Figure 10 illustrate the Exploration Rate of MAPPO, DGR-MAPPO and BFS across maps I, II and III as a function of the number of execution steps.

**Figure 11:**
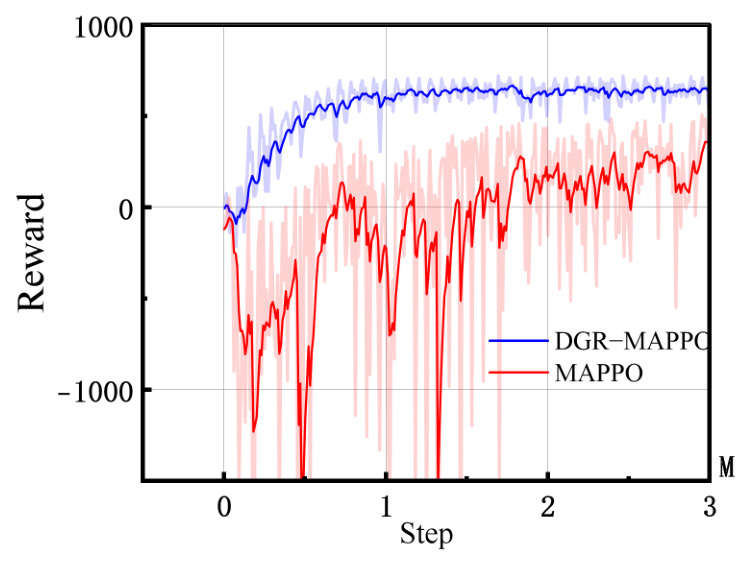
Comparison of training reward curves between standard MAPPO and DGR-MAPPO.

By analyzing the above three experimental result tables and Figure 11, the following conclusions can be drawn:

DGR-MAPPO demonstrates outstanding exploration performance and robustness across various map environments. Compared to the standard MAPPO, DGR-MAPPO significantly surpasses it across all three test maps, achieving average exploration rate improvements of 9.6%, 25.3%, and 34.5%. This performance gap consistently widens with increasing map complexity. The disparity primarily arises from dual enhancements in the feature extraction paradigm and the information-sharing mechanism. By utilizing global average pooling (GAP) instead of fully connected (FC) layers, DGR-MAPPO eliminates millions of training parameters, compelling the model to learn generalized spatial features (“whether a feature exists”) rather than memorizing specific obstacle distributions (“where the feature is located”). Additionally, the distance-based communication mechanism allows agents to instantly share local maps, effectively mitigating the redundant exploration of identical regions commonly seen in the standard MAPPO. In contrast, while the traditional BFS method achieves commendable metrics in certain scenarios (such as the highest peak exploration rate), it fundamentally relies on greedy allocation and static path planning. This deterministic nature lacks the ability to adapt to environmental changes, resulting in lower efficiency and insufficient collaboration in complex or dynamic environments when compared to DGR-MAPPO’s adaptive deep reinforcement learning strategies.

In terms of stability, DGR-MAPPO shows a pronounced advantage. The gap in training stability between DGR-MAPPO and the standard MAPPO is particularly striking: the exploration variance of MAPPO on Map III reaches 0.0157, which is 14.3 times that of DGR-MAPPO (0.0011). More critically, the standard MAPPO experiences severe policy collapse in certain test runs (minimum exploration rate of merely 0.1852), as its feedforward network lacks historical memory and fails to distinguish between “temporary local obstruction” and “exhaustion of global exploration.” DGR-MAPPO overcomes this through the temporal modeling capability of its GRU unit, which encodes trajectory history to provide contextual awareness. This enables effective escape behaviors when agents are confronted with local optima or dead ends, maintaining a minimum exploration rate above 0.8 across all three maps. This highly consistent policy output further highlights its superiority over static methods like BFS, which struggles with limited scalability in large-scale tasks and cannot dynamically resolve complex multi-agent spatial conflicts as effectively as the memory-augmented DGR-MAPPO.

Regarding convergence speed, DGR-MAPPO consistently outperforms its counterparts. The standard MAPPO fails to converge within the 350-step limit on any of the three maps, whereas DGR-MAPPO achieves convergence within 279 to 318 steps. This acceleration is driven by the synergy between the communication mechanism and GAP pooling. The communication module expands the effective field of view for each agent, fundamentally accelerating the aggregation of environmental state information. Simultaneously, GAP prevents the dilution of optimization signals across massive FC parameter spaces, concentrating gradient updates on convolutional kernels and thereby providing the representational foundation for early policy convergence. Furthermore, DGR-MAPPO also outperforms the BFS method in terms of the number of convergence steps. By adaptively adjusting strategies, DGR-MAPPO enables more efficient collaboration and division of labor, accelerating the overall exploration process compared to the generally higher convergence steps required by BFS.

Despite these significant improvements, DGR-MAPPO still has certain limitations. In some extreme map environments, its final average exploration rate can be slightly lower than that of BFS. This indicates that methods based on reinforcement learning may occasionally be affected by training stability and stochastic exploration strategies, resulting in individual experimental outcomes being inferior to those of traditional deterministic methods. Additionally, the implementation and training process of DGR-MAPPO is relatively complex. Compared to heuristic methods, it requires higher computational resources and more intricate parameter tuning. Future work will focus on further optimizing the exploration strategy and training process for extreme scenarios while maintaining the algorithm’s high efficiency in multi-agent collaborative tasks.

### 4.4. The Ablation Experiment of the Global Average Pooling and Fully Connected Layers

**Figure 12:**
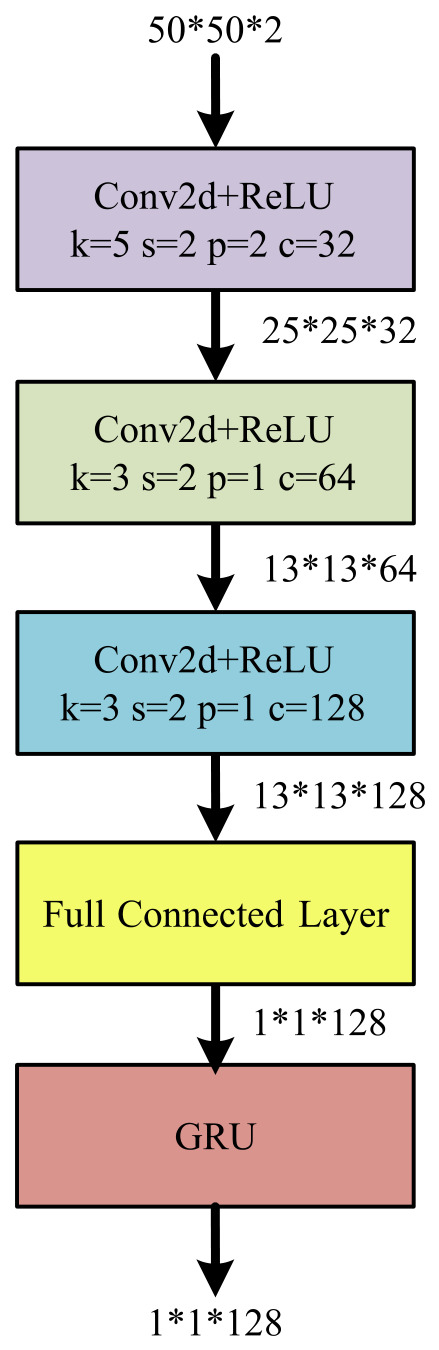
CNN architecture incorporating fully connected networks.

In the feature extraction layer, this study employs a global average pooling layer in place of the original fully connected layer. Ablation experiments were performed on the CNN network structure utilizing the global average pooling layer and the fully connected layer, as depicted in Figure 6 and Figure 12. We only modified the CNN network layer, and the rest were consistent with DGR-MAPPO. The two algorithms were denoted as MAPPO-GAP and MAPPO-FC for comparative purposes. Table 5, Table 6 and Table 7 provide a detailed account of the exploration performance of two distinct algorithm schemes, MAPPO-FC and MAPPO-GAP, across various map environments. Key indicators include the minimum exploration rate, maximum exploration rate, average exploration rate, number of convergence steps, and exploration variance. The average exploration rate and convergence steps are calculated from the data of 20 episodes to ensure both stability and representativeness. The minimum and maximum exploration rates serve to measure the fluctuation range of the algorithms across different experimental runs, thereby reflecting their stability in exploration tasks. Additionally, exploration variance offers insights into the consistency of the algorithms across multiple experiments; a smaller value indicates a more stable exploration effect under varying environments or operating conditions. These experimental data not only illustrate the exploration capabilities of each algorithm in diverse settings but also facilitate comparisons regarding convergence speed, stability, and adaptability.

By analyzing the above three experimental result tables, the following conclusions can be drawn:

1. The exploration capability of MAPPO-GAP is generally superior to that of MAPPO-FC. For instance, as shown in Table 6, the average exploration rate of MAPPO-GAP is 0.9076, significantly exceeding the 0.8127 rate of MAPPO-FC. Additionally, in Table 7, the minimum exploration rate of MAPPO-GAP is 0.832, which is substantially higher than the 0.3573 rate of MAPPO-FC, further demonstrating that MAPPO-GAP exhibits a more stable exploration ability. This advantage arises from global average pooling, which effectively integrates local observation information, thereby enhancing the stability of the agent’s exploration in complex environments and improving the model’s generalization ability. In contrast, MAPPO-FC employs a fully connected layer for feature fusion, which is susceptible to losing spatial information, resulting in diminished generalization capability. Furthermore, the fully connected layer may introduce redundant features when processing high-dimensional data, adversely affecting decision-making accuracy and consequently reducing exploration efficiency.
2. The variance of exploration serves as an indicator of the agent’s stability throughout the experimental process. A smaller variance signifies reduced fluctuations in the agent’s exploration, thereby indicating greater stability. According to the data presented in the table, MAPPO-GAP exhibits the lowest exploration variance (0.0003–0.0011) across all experiments, suggesting that this method demonstrates superior stability and generalization capabilities in various environments. In contrast, MAPPO-FC performs inadequately on untrained maps, reflecting its limited generalization ability. This limitation suggests that the fully connected layer is susceptible to losing spatial location information during the information fusion process. Consequently, this method frequently converges to local optima, resulting in insufficient exploration.

**Figure 13:**
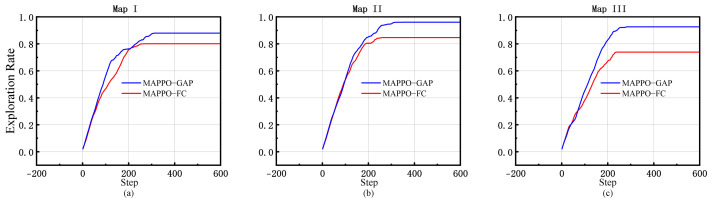
Comparison chart of exploration rates between MAPPO-GAP and MAPPO-FC in different maps. (**a**) Map I; (**b**) Map II; (**c**) Map III.

Figure 13 illustrate the exploration rate of MAPPO-FC and MAPPO-GAP across maps I, II, and III as a function of the number of execution steps.

Notably, MAPPO-GAP (blue curve) demonstrates superior performance, rapidly increasing the exploration rate within a relatively short execution period on each map while maintaining high stability throughout the subsequent training process. This behavior indicates an effective learning speed and a robust collaboration mechanism. In contrast, MAPPO-FC (red curve) exhibits slightly lower exploration efficiency compared to MAPPO-GAP. Importantly, the exploration rate of MAPPO-FC during the later stages of convergence is significantly lower than that of MAPPO-GAP, particularly in tests involving maps not included in the training phase, resulting in a poor final convergence value. This observation suggests that, despite the implementation of multi-agent collaboration, the generalization ability of the fully connected feature fusion (FC) approach is limited when confronted with environmental changes and variations in map layouts. Consequently, this limitation hampers adaptability to the complexities of different environments and leads to diminished exploration capabilities in novel scenarios. Thus, further optimization of feature extraction and fusion mechanisms remains a critical area for research.

### 4.5. The Ablation Experiment of the Communication Mechanisms

This experiment employs a distance-based communication mechanism whereby agents can communicate and exchange local map information when the Euclidean distance between them falls below a specified threshold. Three experimental conditions were established for comparison:

1. No communication: Each agent makes decisions solely based on its own local observations without sharing information with others.
2. Communication distance 25: When the Euclidean distance between two agents is less than 25, they merge their respective local map information, enabling more informed decision-making based on peers’ exploration results.
3. Communication distance 50: When the Euclidean distance between two agents is less than 50, local map information is merged; compared to the distance-25 condition, this allows information sharing over greater distances and enhances collective environmental awareness. This is also the DGR-MAPPO algorithm we proposed.

**Figure 14:**
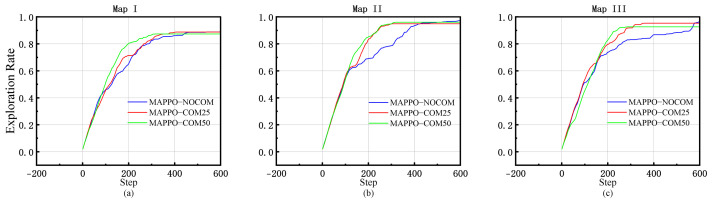
Comparison chart of exploration rates of different communication mechanisms in different maps. (**a**) Map I; (**b**) Map II; (**c**) Map III.

The effects of the three strategies—MAPPO-NOCOM (no communication), MAPPO-COM25 (communication range 25), and MAPPO-COM50 (communication range 50)—are compared in the Figure 14. The trend of the curves indicates that as the number of exploration steps increases, the exploration rates of all three methods gradually rise and ultimately converge. Notably, there are significant differences in exploration efficiency and final convergence values among the strategies employing different communication mechanisms.

The primary distinction among the three experiments is found in the final number of convergence steps, which indicates the influence of various strategies on exploration efficiency. Table 8, Table 9 and Table 10 presents the exploration rates and variance data for the three experiments conducted. The data reveal that the exploration efficiency of the three experiments exhibits minimal differences during the initial phase of exploration. However, as exploration progresses, the areas yet to be explored gradually diminish. The agent lacking communication is unable to recognize the regions that other agents have already explored. In contrast, the experiment that incorporates a communication mechanism demonstrates that information sharing among agents can significantly enhance convergence speed. As the communication range expands, the thoroughness of information sharing among agents increases, further improving the efficiency of strategy learning. Concurrently, this facilitates optimized action planning, resulting in a more coordinated and efficient exploration strategy for the entire system.

**Figure 15:**
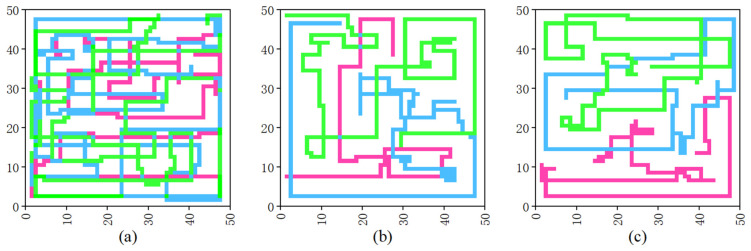
Comparison chart of path differences for different communication mechanisms. The red, blue, and green lines represent the paths of Agent 1, Agent 2, and Agent 3, respectively. (**a**) MAPPO-NOCOM; (**b**) MAPPO-COM25; (**c**) MAPPO-COM50.

Figure 15 illustrate the exploration paths of the three agents across the three experimental settings. In each figure, trajectories represented in various colors correspond to the movement paths of distinct agents, visually highlighting the differences in exploration behaviors under varying communication conditions.

In the MAPPO-NOCOM experiment, the absence of a communication mechanism resulted in the three agents making decisions and acting entirely independently, without sharing information. This independence led to significant path overlap and pronounced regional redundancy during the exploration process. Consequently, this lack of a collaborative exploration approach diminished overall exploration efficiency and resulted in resource wastage.

In the MAPPO-COM25 experiment, the communication radius of the agent was established at 25 unit lengths, enabling the exchange of positional information within a specified range. The implementation of various communication mechanisms facilitated initial collaboration among agents, which significantly diminished the occurrence of path overlap. Consequently, the area explored was more extensive than in scenarios devoid of communication, leading to a marked enhancement in overall exploration efficiency. Furthermore, the division of labor and coordination among agents began to manifest progressively.

In the MAPPO-COM50 experiment, the communication radius was increased to 50 unit lengths, resulting in more frequent and effective information exchange among agents. This enhancement allowed each agent to more effectively plan its exploration trajectory and actively avoid areas previously covered by other agents, thereby reducing the path overlap rate. Under these experimental conditions, the overall agent system exhibited the highest exploration efficiency and optimal resource utilization, thereby confirming the substantial role of improved communication capabilities in facilitating collaborative exploration tasks among multiple agents.

**Figure 16:**
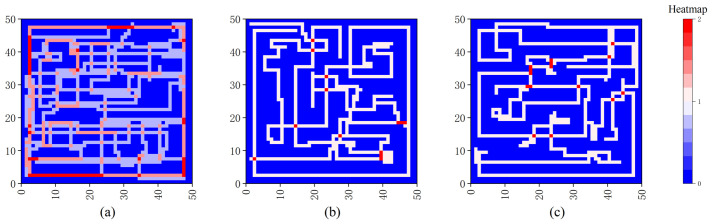
Comparison chart of path heat maps for different communication mechanisms. (**a**) MAPPO-NOCOM; (**b**) MAPPO-COM25; (**c**) MAPPO-COM50.

Figure 16 illustrate the path heat maps of the three agents across various experimental settings. A comparison of these heat maps reveals the impact of different communication mechanisms on the exploration behavior of the agents. In the absence of a communication mechanism, the agent exhibits a relatively high path repetition rate, suggesting a lack of information sharing during the exploration process. This results in considerable redundant exploration and diminishes overall exploration efficiency. Conversely, the introduction of information sharing in the MAPPO-COM25 and MAPPO-COM50 experiments leads to a significant reduction in path repetition rates. This finding indicates that the agents can effectively exchange environmental information through the communication mechanism, thereby optimizing their exploration strategies. As the communication range increases, the degree of collaboration among agents is further enhanced, leading to a more uniform distribution of exploration areas and a marked decrease in repetitive exploration.

In Experiment Three (MAPPO-COM50), the agent’s path heat map exhibited significantly fewer path repetitions, and the number of convergence steps was markedly lower than that observed in Experiment Two (MAPPO-COM25). This finding suggests that as communication distance increases, the frequency and timeliness of information exchange among agents improve, thereby reducing redundant exploration and enhancing exploration efficiency. An increased communication distance allows each agent to perceive and share environmental information over a broader range, enabling individuals to consider a more comprehensive array of local and global information when making decisions, thus optimizing their action strategies. In comparison to a shorter communication range, a longer communication distance permits agents to detect the exploration paths of their peers earlier and adjust their behaviors accordingly to avoid redundant exploration, thereby expediting the global coverage process. Furthermore, a longer communication distance significantly enhances exploration efficiency, ensuring that each round of exploration proceeds more stably. In contrast, experiments lacking communication or employing short communication distances frequently result in repetitive exploration due to delayed communication.

While extended communication distances offer clear benefits for enhancing exploration, they also introduce significant computational demands and resource consumption. As the communication range increases, agents must manage a greater volume of information exchange and processing, which consequently elevates the computational burden on the system. In practical applications, optimizing the allocation of computational resources, while maintaining communication efficiency and minimizing unnecessary computational overhead, emerges as a critical research focus for enhancing the system’s overall performance.

This section presents a comprehensive examination of the exploration processes across three experiments conducted at varying training time steps, along with a visual analysis of the agent’s environmental map. By comparing the exploration paths and coverage areas of the agent under different experimental settings, one can visually discern the evolution of the agent’s exploration progress and its adaptability to the environment as training advances. Furthermore, an analysis of the exploration methods employed under diverse experimental conditions will elucidate the role of the communication mechanism in multi-agent collaborative exploration, as well as its effects on exploration efficiency, path optimization, and task completion speed.

**Figure 17:**
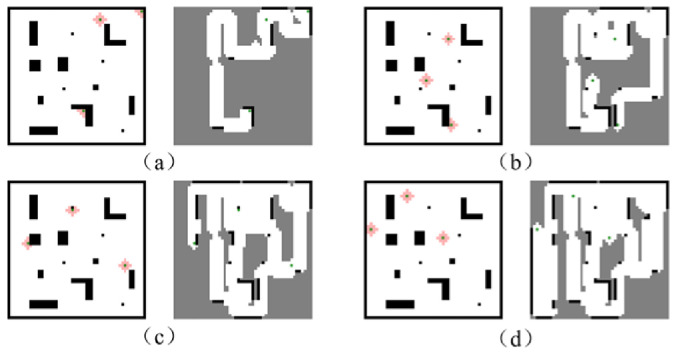
MAPPO-NOCOM Exploration Progress Chart. (**a**) 60 steps; (**b**) 120 steps; (**c**) 180 steps; (**d**) 240 steps. Black: obstacles; white: explored areas; gray: unexplored areas; green dot: agent’s current position; red area: perception range.

**Figure 18:**
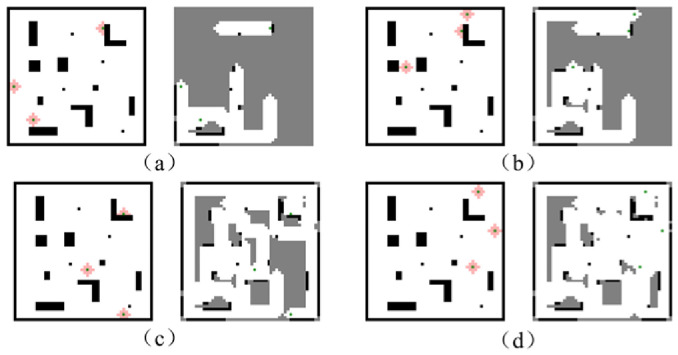
MAPPO-COM25 Exploration Progress Chart. (**a**) 60 steps; (**b**) 120 steps; (**c**) 180 steps; (**d**) 240 steps. Black: obstacles; white: explored areas; gray: unexplored areas; green dot: agent’s current position; red area: perception range.

**Figure 19:**
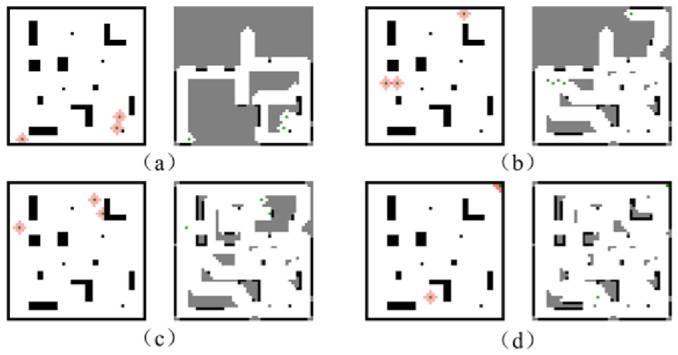
MAPPO-COM50 Exploration Progress Chart. (**a**) 60 steps; (**b**) 120 steps; (**c**) 180 steps; (**d**) 240 steps. Black: obstacles; white: explored areas; gray: unexplored areas; green dot: agent’s current position; red area: perception range.

Figure 17, Figure 18 and Figure 19 depict the environmental maps and corresponding exploration scenarios for the three experiments at various time steps (60, 120, 180, and 240 steps) on the untrained map (corresponding to Figures a, b, c, and d). These graphs clearly illustrate that, in the absence of information sharing, the agent’s exploration efficiency is relatively low, the coverage of the exploration area is insufficient, and there is considerable redundancy. Conversely, in the experiments that incorporate communication mechanisms, an increase in communication distance leads to more frequent and effective information sharing among agents, resulting in a more coordinated and efficient exploration process.

**Figure 20:**
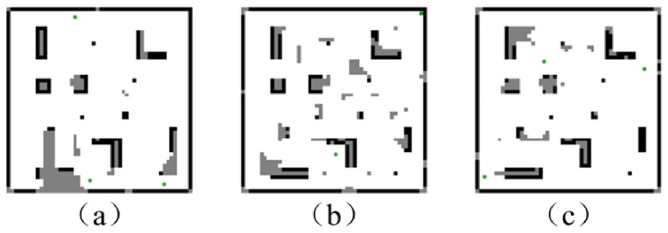
The final environmental exploration results of different communication mechanisms. (**a**) MAPPO-NOCOM; (**b**) MAPPO-COM25; (**c**) MAPPO-COM50. Green dot: agent’s current position.

Figure 20 presents the final environmental exploration results of the three experiments (from left to right: MAPPO-NOCOM, MAPPO-COM25, and MAPPO-COM50), with gray areas denoting unexplored regions. The data reveal that the completion rate of environmental exploration improves with the introduction of information sharing. Notably, when the communication range is set to 50, the environmental coverage rate achieves its peak. This finding suggests that an effective communication mechanism can significantly enhance the collaborative exploration capabilities of multi-agent systems and expedite task completion.

### 4.6. The Ablation Experiment of the Gated Recurrent Unit

**Figure 21:**
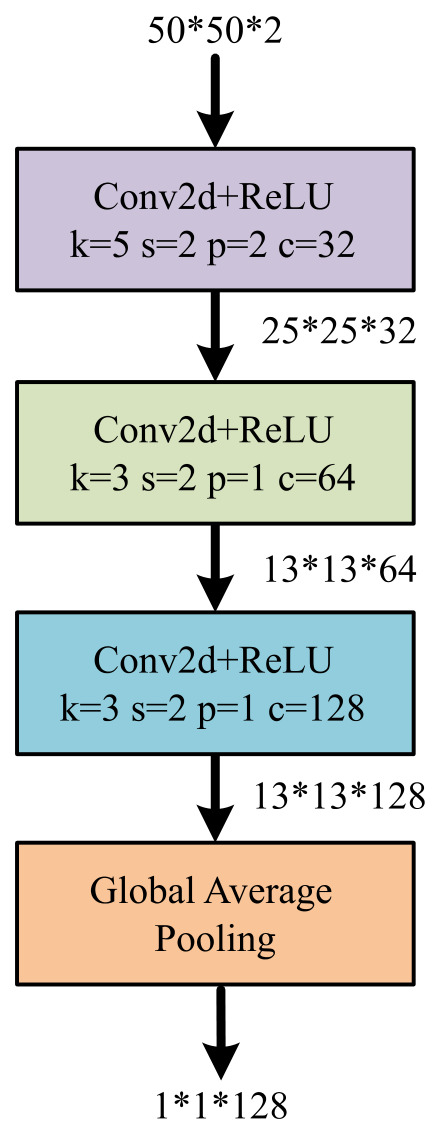
CNN architecture excluding GRU units.

To assess the influence of the gated recurrent unit (GRU) on model performance, DGR-MAPPO was designated as the control group for this experiment. The CNN network without using GRU is shown in Figure 21. Consistency in experimental conditions was maintained, with the reward function, observation space, action space, and hyperparameter settings remaining unchanged. The sole distinction involved the feature extraction network design: the experimental group incorporated an additional GRU layer into the original feature extraction network to enhance the agent’s capacity to model temporal information, thereby improving the efficiency and stability of the exploration task. This experimental group was subsequently referred to as MAPPO-GRU for comparative analysis with MAPPO-NOGRU.

**Figure 22:**
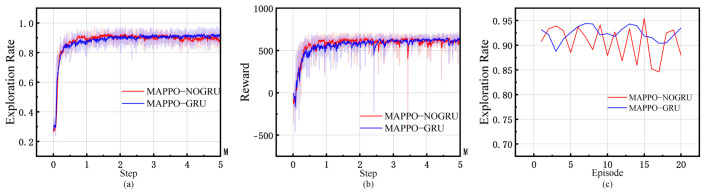
Comparison chart of the training process with or without the addition of GRU based on different indicators in (**a**,**b**). Experiment 20-round exploration rate comparison chart in (**c**). (**a**) Exploration rate comparison, (**b**) reward value comparison, and (**c**) exploration rate comparison chart.

During the experiment, the agent records its exploration rate and reward value in the current state every 15 training rounds. These data facilitate the monitoring of the agent’s training convergence trend and the evaluation of the model’s overall performance. As shown in Figure 22a,b, analysis of the curves depicting exploration rate and reward variation reveals that, although the final difference in exploration rates is minimal, the introduction of the GRU module into the feature extraction network enhances the stability of the model’s training process, significantly reducing the amplitude of fluctuations. This finding suggests that the GRU effectively models temporal information, thereby improving the stability and convergence of the training process.

To further assess the generalization capability of the model, Map, which was not included in the training dataset, was selected for testing. The trained model underwent 20 rounds of evaluation, during which the exploration rate of the agent was recorded in each round to evaluate the adaptability of different models in novel environments. Figure 22c illustrates the variations in the exploration rates of MAPPO-NOGRU and MAPPO-GRU throughout the 20 rounds of testing. Notably, the MAPPO-GRU model, which incorporates the GRU module, exhibited greater stability during the exploration process, with a significantly reduced range of fluctuation in the exploration rate. Experimental results indicate that the GRU module enhances the continuity of decision-making and the adaptability of the agent to its environment by effectively modeling temporal information. This improvement leads to a more stable exploration strategy, thereby augmenting the agent’s generalization ability in unfamiliar environments.

### 4.7. The Ablation Experiment of the Number of Agents

We examine how exploration performance varies with the number of agents N∈{2,3,4,5,6}. For each *N*, a MAPPO policy is independently trained from scratch with identical hyperparameters, and evaluated on three maps (Map I/II/III) over 20 episodes of 400 steps. Four metrics are reported: average, maximum, and minimum exploration rate; convergence steps—the mean over 20 episodes of the step at which each episode’s exploration-rate curve enters its plateau region (the final stable segment); and path overlap rate—the fraction of grid cells traversed by two or more agents. The full results are summarized in Table 11, Table 12 and Table 13.

The exploration rate exhibits a sharp saturation at N=3 that is consistent across all three maps. The mean marginal gain from N=2 to N=3 is +0.113, but collapses to +0.005 from N=3 to N=4—a twenty-fold reduction. On Map I, the 3→4 gain is a negligible +0.002; on Map III, it is only +0.0003. For N≥4, the per-agent gain remains below +0.011, indicating that additional agents contribute little new coverage beyond the three-agent configuration.

Convergence speed, however, continues to benefit from larger agent counts. The convergence steps decrease monotonically from 391 (N=2) to 266 (N=6), averaged across three maps, yielding a cumulative speedup of 1.47×. The per-step contribution follows a steeply diminishing pattern: the 2→3 transition alone accounts for 90 of the total 125 steps saved, while the 3→4 transition saves only 7 steps. This decoupling—exploration rate flatlines while convergence keeps accelerating—reveals a functional role shift: agents added beyond N=3 primarily improve convergence efficiency rather than the final coverage ceiling.

The cost of adding agents manifests in the path overlap rate, which rises monotonically from 1.1% (N=2) to 5.9% (N=6) averaged across maps. Each additional agent contributes a nearly constant +0.010∼0.014 to the overlap rate, indicating that inter-agent interference accumulates linearly rather than accelerating. The exploration spread (max−min) further corroborates the saturation narrative: on Map III, it drops sharply from 0.424 at N=2 to 0.108 at N=3, and continues declining to 0.051 at N=6. The largest stability gain coincides precisely with the saturation point, after which run-to-run variability remains low.

Taken together, these four dimensions delineate a two-phase degradation law. In the gain phase (N≤3), each added agent substantially improves coverage (+0.113/agent), accelerates convergence (30% speedup from 2→3), and stabilizes behavior (63% spread reduction). In the saturation phase (N≥4), exploration gain drops below +0.011/agent while path overlap continues its linear accumulation. The saturation point $N^{*}\approx 3$ is observed across all three structurally distinct test maps and all four metrics, establishing it as an intrinsic property of DGR-MAPPO’s pairwise communication mechanism rather than a map-specific artifact.

### 4.8. Zero-Shot Scaling to 100 × 100 Maps

**Figure 23:**
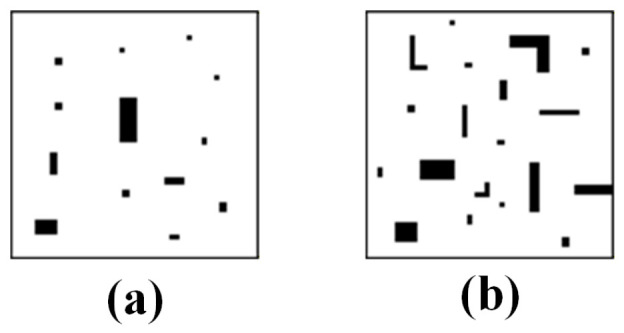
Two unseen 100×100 maps. (**a**) Simple scenario, denoted as Map IV; (**b**) complex scenario, denoted as Map V.

To evaluate whether the learned cooperative exploration policy generalizes to substantially larger environments, we conduct zero-shot transfer experiments on 100×100 grid worlds, a fourfold increase in area compared to the 50×50 maps (2500 vs. 10,000 cells) used during training. The pretrained model is deployed without any fine-tuning or architectural modification: the CNN backbone in the policy network employs adaptive average pooling, which naturally accepts variable spatial input dimensions. Two previously unseen 100×100 maps with distinct obstacle layouts are used, as shown in Figure 23. Map IV features relatively sparse obstacles with wide open corridors, providing a simpler exploration landscape, while Map V features denser and more fragmented obstacle distributions, posing a greater challenge for complete coverage. On each map, we perform 20 independent evaluation episodes of 1000 steps with three agents, matching the training configuration in all respects except map size.

As can be seen from Table 14, the model achieves a mean exploration rate of 0.819 over 20 runs on Map IV, with the best episode reaching 0.881 coverage of the 10,000-cell environment. On Map V, the mean is 0.687, with a best-case of 0.827. Across all 40 episodes (20 per map), the policy consistently achieves substantially higher exploration rates than a random baseline, demonstrating that the cooperative strategy learned on small maps transfers effectively.

**Figure 24:**
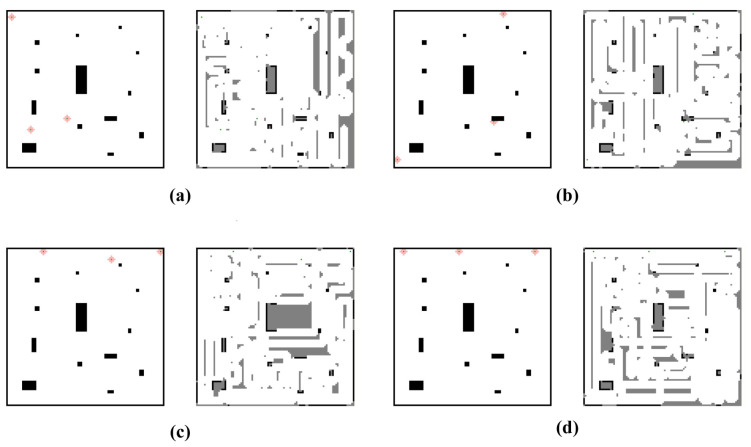
In Map IV, the final exploration maps on 100×100 grid worlds. Each image shows the ground-truth occupancy grid (**left**) and the explored area with agent positions (**right**). (**a**–**d**) represent the final results of the four random rounds. Red area: perception range.

**Figure 25:**
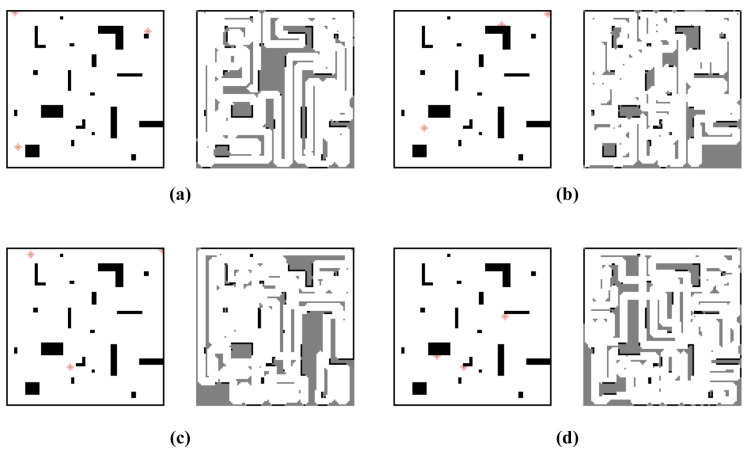
In Map V, the final exploration maps on 100×100 grid worlds. Each image shows the ground-truth occupancy grid (**left**) and the explored area with agent positions (**right**). (**a**–**d**) represent the final results of the four random rounds. Red area: perception range.

Figure 24 and Figure 25 respectively present the randomly generated final exploration maps on maps IV and V. The lower left part of each image represents the actual occupied grid, while the lower right part shows the areas discovered by the agent (white indicates the explored and free space, black indicates obstacles, and gray indicates unknown areas).

Three observations merit discussion. First, the model transfers to a 4× larger environment without retraining, indicating that the learned exploration behavior is not memorization of specific training maps but a generalizable cooperative strategy. The CNN backbone’s adaptive pooling eliminates the need to resize or interpolate the agent’s observation, preserving the spatial fidelity of the input.

Second, the performance gap between Map IV and Map V (approximately 13 percentage points in mean exploration rate) demonstrates sensitivity to obstacle layout complexity. Map V’s denser or more fragmented obstacle distribution likely creates more narrow corridors and dead ends, increasing the difficulty of complete exploration for a fixed-step budget. This variability is a feature rather than a flaw: it shows the evaluation is not confined to a single favorable map.

Third, the variance of the final exploration rate across 20 runs (0.0025 on Map IV and 0.0045 on Map V) is moderate, indicating that while the policy reliably explores a large fraction of the environment, the precise coverage depends on the stochastic initial positions of the agents. This variability is consistent with the behavior observed on 50×50 training maps and is inherent to the decentralized exploration paradigm.

## 5. Conclusions and Future Work

This paper investigates a multi-agent collaborative exploration method for unknown grid environments, grounded in the MAPPO algorithm and the CTDE paradigm, and proposes the DGR-MAPPO algorithm to address the dual challenges of exploration efficiency and dependence on global information. The core contributions are threefold: a distance-aware communication mechanism that enables agents within communication range to exchange information and incrementally update their local maps; a feature extraction network that employs global average pooling (GAP) in place of fully connected layers and integrates a gated recurrent unit (GRU) for temporal modeling; and a dual-channel image-fusion observation environment that synchronizes global exploration state with local perception details. Crucially, these three modules are not simple superposition but form a closed-loop synergy: the communication mechanism expands the effective observation field, providing richer spatial input for GAP-based feature aggregation; GAP compresses the parameter space and concentrates gradients on convolutional kernels, enabling efficient encoding of the communication-augmented observations; and GRU encodes trajectory history to provide contextual memory when communication is constrained or agents fall into local optima, compensating for the temporal information that spatial pooling alone cannot capture. This “information acquisition → feature compression → temporal memory” closed loop distinguishes DGR-MAPPO from a conventional engineering stack of independent optimizations.

Ablation experiments confirm the individual contribution of each module. Replacing fully connected layers with GAP substantially reduces the parameter count while preserving exploration rate and improving generalization across maps, confirming that spatial feature aggregation is more effective than positional memorization for exploration tasks. The communication mechanism ablation shows that exploration rate improves and path overlap declines as the communication distance increases, with a clear saturation trend that delimits the effective range of information sharing. The GRU ablation demonstrates that recurrent temporal modeling markedly enhances training stability and mitigates policy collapse, validating the necessity of historical context under partial observability.

Comparative and scaling experiments further validate the full DGR-MAPPO design. Against the standard MAPPO baseline, DGR-MAPPO achieves average exploration-rate improvements of 9.6%, 25.3%, and 34.5% on the three test maps, with the gap widening as map complexity increases; the standard MAPPO even suffers policy collapse on Map III (minimum exploration rate of only 0.1852), while DGR-MAPPO maintains a minimum above 0.8 across all maps. These results confirm that the performance gains originate from the complete DGR-MAPPO design rather than from the MAPPO framework itself. The ablation on the number of agents reveals a two-phase degradation law: a gain phase for N≤3 with a marginal improvement of +0.113 per agent, and a saturation phase for N≥4 where the per-agent gain drops below +0.011 while path overlap accumulates linearly. The saturation point $N^{*}\approx 3$ is consistent across three structurally distinct maps, establishing it as an intrinsic property of the pairwise communication mechanism. Zero-shot transfer to 100 × 100 maps—a fourfold increase in area—achieves mean exploration rates of 0.819 and 0.687 on two unseen maps, demonstrating that the learned policy is a generalizable cooperative strategy rather than a memorization of training layouts. Compared with the BFS-based frontier method, DGR-MAPPO converges in fewer steps and adapts better to complex layouts, though its average exploration rate remains 5–8% lower in extreme deterministic scenarios, reflecting the inherent trade-off between adaptive learning and deterministic planning.

Despite these results, the proposed method has clear limitations and applicable boundaries. The agent-count study shows that inter-agent interference accumulates linearly once *N* exceeds the saturation point, indicating an upper bound on the redundancy-suppression capability of the pairwise communication mechanism. On larger 10,000-cell maps, the convergence steps approach the 1000-step budget, suggesting that the exploration policy has not fully saturated within the step limit. Regarding physical deployment, the method is general and can be deployed on any robot equipped with a two-dimensional lidar; however, the kinematic structure (omnidirectional versus differential-drive) must be considered, and fine-tuning of the policy is required at deployment time. Additionally, the current evaluation is confined to simulation and static obstacles, and real-world validation has not yet been conducted.

Future work will focus on the following directions. First, extending the method to dynamic environments with moving obstacles and time-varying topologies, and studying the robustness of the distance-aware communication mechanism under such perturbations. Second, optimizing the communication mechanism itself—exploring sparse, prioritized, or learnable communication ranges to reduce path overlap when *N* exceeds the saturation point. Third, investigating long-horizon and hierarchical exploration strategies, including sub-goal decomposition and explore-exploit adaptive scheduling, to address the step-budget limitation observed on large maps. Fourth, developing meta-learning approaches that dynamically adjust the number of active agents based on map complexity, building on the observed $N^{*}\approx 3$ saturation law. As multi-agent reinforcement learning continues to mature, the DGR-MAPPO framework is expected to facilitate the broader deployment of agent collaboration technology in scenarios such as disaster search and rescue, industrial facility inspection, and large-scale warehouse logistics.

## Figures and Tables

**Table 1:** Hyperparameter settings for MAPPO experiments.

Parameter	Value
n_rollout_threads	5
actor_lr	0.0005
critic_lr	0.0005
num_env_steps	1,000,000
episode_length	400
gamma	0.99
ppo_epoch	15
num_mini_batch	2
clip_param	0.2
gae_lambda	0.95

**Table 2:** MAPPO experimental results comparison (Map I).

Algorithm	Min Exp Rate	Max Exp Rate	Ave Exp Rate	Conv Steps	Exp Variance
BFS	0.9028	0.9028	0.9028	785	0.0000
DGR-MAPPO	0.816	0.8636	0.8389	279	0.0003
MAPPO	0.6844	0.7976	0.7657	423	0.0007

**Table 3:** MAPPO experimental results comparison (Map II).

Algorithm	Min Exp Rate	Max Exp Rate	Ave Exp Rate	Conv Steps	Exp Variance
BFS	0.9876	0.9876	0.9876	749	0.0000
DGR-MAPPO	0.832	0.9448	0.9074	307	0.0011
MAPPO	0.6604	0.814	0.7239	397	0.0008

**Table 4:** MAPPO experimental results comparison (Map III).

Algorithm	Min Exp Rate	Max Exp Rate	Ave Exp Rate	Conv Steps	Exp Variance
BFS	0.958	0.958	0.958	575	0.0000
DGR-MAPPO	0.846	0.9544	0.9069	318	0.0011
MAPPO	0.1852	0.7644	0.6743	402	0.0157

**Table 5:** Ablation Study on the Impact of Global Average Pooling and Fully Connected Layers on MAPPO Performance (Map I).

Algorithm	Min Exp Rate	Max Exp Rate	Ave Exp Rate	Conv Steps	Exp Variance
MAPPO-FC	0.6589	0.7921	0.7378	254	0.0011
MAPPO-GAP	0.816	0.8636	0.8389	279	0.0003

**Table 6:** Ablation Study on the Impact of Global Average Pooling and Fully Connected Layers on MAPPO Performance (Map II).

Algorithm	Min Exp Rate	Max Exp Rate	Ave Exp Rate	Conv Steps	Exp Variance
MAPPO-FC	0.7078	0.8574	0.8127	259	0.0018
MAPPO-GAP	0.832	0.9448	0.9076	307	0.0011

**Table 7:** Ablation Study on the Impact of Global Average Pooling and Fully Connected Layers on MAPPO Performance (Map III).

Algorithm	Min Exp Rate	Max Exp Rate	Ave Exp Rate	Conv Steps	Exp Variance
MAPPO-FC	0.3573	0.8167	0.6165	235	0.0311
MAPPO-GAP	0.832	0.9448	0.9074	307	0.0011

**Table 8:** Performance comparison of MAPPO under different communication ranges (Map I).

Algorithm	Min Exp Rate	Max Exp Rate	Ave Exp Rate	Conv Steps	Exp Variance
MAPPO-NOCOM	0.7172	0.8888	0.8634	461	0.0019
MAPPO-COM25	0.7652	0.8584	0.8251	405	0.0019
MAPPO-COM50	0.816	0.8636	0.8389	279	0.0003

**Table 9:** Performance comparison of MAPPO under different communication ranges (Map II).

Algorithm	Min Exp Rate	Max Exp Rate	Ave Exp Rate	Conv Steps	Exp Variance
MAPPO-NOCOM	0.8772	0.9736	0.9394	594	0.0011
MAPPO-COM25	0.806	0.9436	0.8991	300	0.0017
MAPPO-COM50	0.832	0.9448	0.9074	307	0.0011

**Table 10:** Performance comparison of MAPPO under different communication ranges (Map III).

Algorithm	Min Exp Rate	Max Exp Rate	Ave Exp Rate	Conv Steps	Exp Variance
MAPPO-NOCOM	0.716	0.9724	0.8628	530	0.0101
MAPPO-COM25	0.8188	0.9588	0.8912	361	0.0019
MAPPO-COM50	0.846	0.9544	0.9069	318	0.0011

**Table 11:** Comparison of results based on different numbers of intelligent agents (Map I).

Num	Min Exp Rate	Max Exp Rate	Ave Exp Rate	Conv Steps	Avg Path Overlap Rate
2	0.6576	0.8402	0.7503	386	0.0115
3	0.816	0.8636	0.8389	279	0.0219
4	0.8192	0.8678	0.8406	281	0.0315
5	0.8022	0.8692	0.8443	271	0.0373
6	0.8212	0.8648	0.8522	254	0.0476

**Table 12:** Comparison of results based on different numbers of intelligent agents (Map II).

Num	Min Exp Rate	Max Exp Rate	Ave Exp Rate	Conv Steps	Avg Path Overlap Rate
2	0.7944	0.909	0.8538	375	0.0146
3	0.832	0.9448	0.9074	307	0.0289
4	0.8974	0.9518	0.9201	301	0.0392
5	0.9134	0.9536	0.93	282	0.0524
6	0.9152	0.9562	0.9368	269	0.0646

**Table 13:** Comparison of results based on different numbers of intelligent agents (Map III).

Num	Min Exp Rate	Max Exp Rate	Ave Exp Rate	Conv Steps	Avg Path Overlap Rate
2	0.4182	0.8418	0.7087	412	0.0066
3	0.846	0.9544	0.9069	318	0.0203
4	0.8544	0.9414	0.9072	300	0.0361
5	0.8642	0.9546	0.9141	298	0.0477
6	0.8974	0.9488	0.9308	276	0.0657

**Table 14:** Zero-shot transfer results on 100×100 maps (20 runs per map).

	Map IV	Map V
Ave Exp Rate	0.8188	0.6866
Max Exp Rate	0.8812	0.8265
Min Exp Rate	0.7119	0.5855
Conv Steps	959	923
Exp Variance	0.0025	0.0045

## Data Availability

The datasets used and/or analysed during the current study available from the corresponding author on reasonable request. Data requests can be made via email (xachen@cjlu.edu.cn).
